# Does Conventional Narrow-Band Imaging-Guided Biopsy Increase *Helicobacter Pylori* Detection in Day-to-Day Practice?

**DOI:** 10.34172/mejdd.3412

**Published:** 2026-04-30

**Authors:** Tata Ramya Sree, Ganaraj Kulamarva, Shama Prakash Kabekkodu, Prathibha Shankarana Narayana Bhat

**Affiliations:** ^1^Department of General Medicine, Pinameneni Siddartha Institute of Medical Sciences and Research Foundation (PSIMS & RF), Vijayawada, India; ^2^Department of Gastroenterology, Father Muller’s Medical College, Mangaluru, India; ^3^Nitte (Deemed to be University), KS Hegde Medical Academy (KSHEMA), Department of General Medicine, Mangalore, India; ^4^Department of General Medicine, Father Muller’s Medical College, Mangaluru, India

**Keywords:** *Helicobacter pylori*, Narrow band imaging, Gastrointestinal endoscopy, Image-guided biopsy, Rapid urease test

## Abstract

**Introduction:**

Conventional narrow-band imaging-guided site-specific biopsy enhances mucosal visualization in the evaluation of *Helicobacter pylori*, but its concordance with conventional white light endoscopy remains unclear. This study aims to compare the positivity yield and concordance between conventional white-light endoscopy and conventional narrow-band imaging for detecting *Helicobacter pylori* in patients with dyspepsia using the rapid urease test.

**Methods:**

This observational study included 185 adults with dyspeptic symptoms. Gastric biopsies were obtained using white light endoscopy and conventional narrow band imaging, and all specimens were tested for *Helicobacter pylori* using the rapid urease test.

**Results:**

The conventional rapid urease test was positive in 175 patients, and the conventional narrow-band imaging rapid urease test was positive in 172 patients, demonstrating a significant association between the two diagnostic approaches. *Helicobacter pylori* was detected in conventional narrow-band imaging types 1, 2, 3, and 4 in 80%, 93.3%, 97.1%, and 91.4% of cases, respectively; type 5 was absent. No statistically significant association was found between conventional narrow band imaging mucosal patterns and the rapid urease test (*P*<0.001).

**Conclusion:**

Conventional narrow-band imaging-guided biopsy showed positivity yields that were concordant with those of conventional white-light endoscopy-guided biopsy in identifying *Helicobacter pylori* using the rapid urease test. No statistically significant difference in the rapid urease test positivity was observed between the two methods in this study population.

## Introduction

 Dyspepsia, often termed “indigestion” by the general population, is one of the most frequent gastrointestinal complaints encountered by physicians in routine practice. It comprises a group of symptoms such as epigastric pain and burning, early satiety, postprandial fullness, nausea, belching, and anorexia.^1^ Almost 25% of dyspepsia cases are attributed to *Helicobacter pylori* (*H. pylori*).

*H. pylori* infection can cause persistent inflammation of the gastric mucosa, which may progress to peptic ulcer and even gastric cancer.^2^ *H. pylori* is classified as a Class I carcinogen by the World Health Organization (WHO). Guidelines recommend that *H. pylori* infection be screened for and treated as early as possible in high-risk communities.^3^ Hence, accurate detection of *H. pylori* is crucial for diagnosing and managing dyspeptic diseases.

 Histopathology is traditionally regarded as the gold standard due to its accuracy. However, mere visualization of curved bacilli does not always confirm *H. pylori* infection.^4^ Therapid urease test (RUT), which detects urease enzyme in biopsy samples, has its advantages over histopathology as it is simple, inexpensive, and results are obtained rapidly.^5^ Some studies have found no significant differences in *H. pylori* detection between histopathology and RUT.^6^

 Conventional white light endoscopy (WLE) lacks sufficient mucosal specificity for *H. pylori* detection, necessitating biopsy for further confirmation.^7^ Narrow Band Imaging (NBI) is a combination of an electronic endoscope and a source of light equipped with special filters, which has emerged as an accurate non-invasive test to detect *H. pylori* gastritis.^8^ Conventional-NBI (C-NBI) is more widely available and cheaper than high-resolution NBI gastroscopes and is also accurate in the identification of *H. pylori* infection.^9^ With a scarcity of literature in the Indian context, the present study was conducted to compare the positivity yield and concordance of conventional white light endoscopy and site-specific conventional narrow band imaging in the detection of *H. pylori* from gastric biopsies of patients with dyspepsia using RUT. The null hypothesis is that there is no difference in the positivity yield and concordance of C-NBI and conventional WLE.

## Materials and Methods

###  Study Design and Setting

 This observational study was conducted in the Department of General Medicine and Gastroenterology at KS Hegde Medical Academy, Mangaluru, India. The study is reported using the Strengthening the Reporting of Observational Studies in Epidemiology (STROBE) guidelines.

 Ethical clearance (EC/09/2018-19) from the Institutional Ethics Committee was obtained for the study, and the study is in accordance with the ethical principles for medical research involving human subjects outlined in the Declaration of Helsinki (revised 2013). Prior to commencement, written informed consent was obtained from all the participants.

###  Sample Size

 The sample size was calculated using the standard formula for estimating population proportion

 N = Z_1-a/2_/d x P x Q

 Where:

- N = sample size
- Z_1-a/2_ = Standard normal variant (1.96 for 95% confidence level)
- d = Absolute marginal error (5%)
- *P* = Expected prevalence (62%; 0.62)
- Q = 1-P (0.38)

Using an absolute marginal error of 5%, the initial sample size was calculated as 365 participants, then recalculated to 185 participants with a 7% absolute error due to feasibility constraints during the COVID–19 pandemic.

###  Study Participants

 A total of 185 patients who visited the department of General Medicine and Gastroenterology from January 2019 to June 2020 were included in this study.

###  Inclusion Criteria:

a) Patients aged 18 years or above.
b) Patients reporting dyspeptic symptoms.
c) Patients scheduled for upper gastrointestinal endoscopy.
d) Patients willing to participate and provide informed consent.

###  Exclusion Criteria:

a) Patients unwilling to participate or provide consent.
b) Severely ill or medically unstable patients
c) Non-cooperative patients
d) Patients in whom endoscopy was contraindicated.

###  Data Collection

 A detailed history was obtained from the patients, and a thorough examination was conducted for each participant. All the findings were systematically entered into a pre-designed proforma.

###  Endoscopic Evaluation

 None of the participants underwent a proton pump inhibitor (PPI) washout period prior to endoscopy. All enrolled patients underwent standardWLE (Olympus 170 Scope), and endoscopic findings were recorded. Biopsies were taken from the gastric antrum and body using standard WLE. In the same sitting, the patients were examined using C-NBI by pressing a button on the Olympus 170 scope, which activated the NBI light. The appearance of the stomach under C-NBI was noted, and the various C-NBI patterns were identified. Prior to the commencement of the study, the endoscopists were trained and calibrated to recognize C-NBI mucosal patterns. Ambiguous results were resolved through discussion to minimize observer variability.

###  C-NBI Patterns

 The different types of C-NBI patterns (Figure 1) were as follows:

- Type 1: Regularly arranged venules
- Type 2: Gastric pits - Cone shape
- Type 3: Gastric pits - Rod shape
- Type 4: Ground glass pattern
- Type 5: Brownish patches with irregular bluish margin

**Figure 1:**
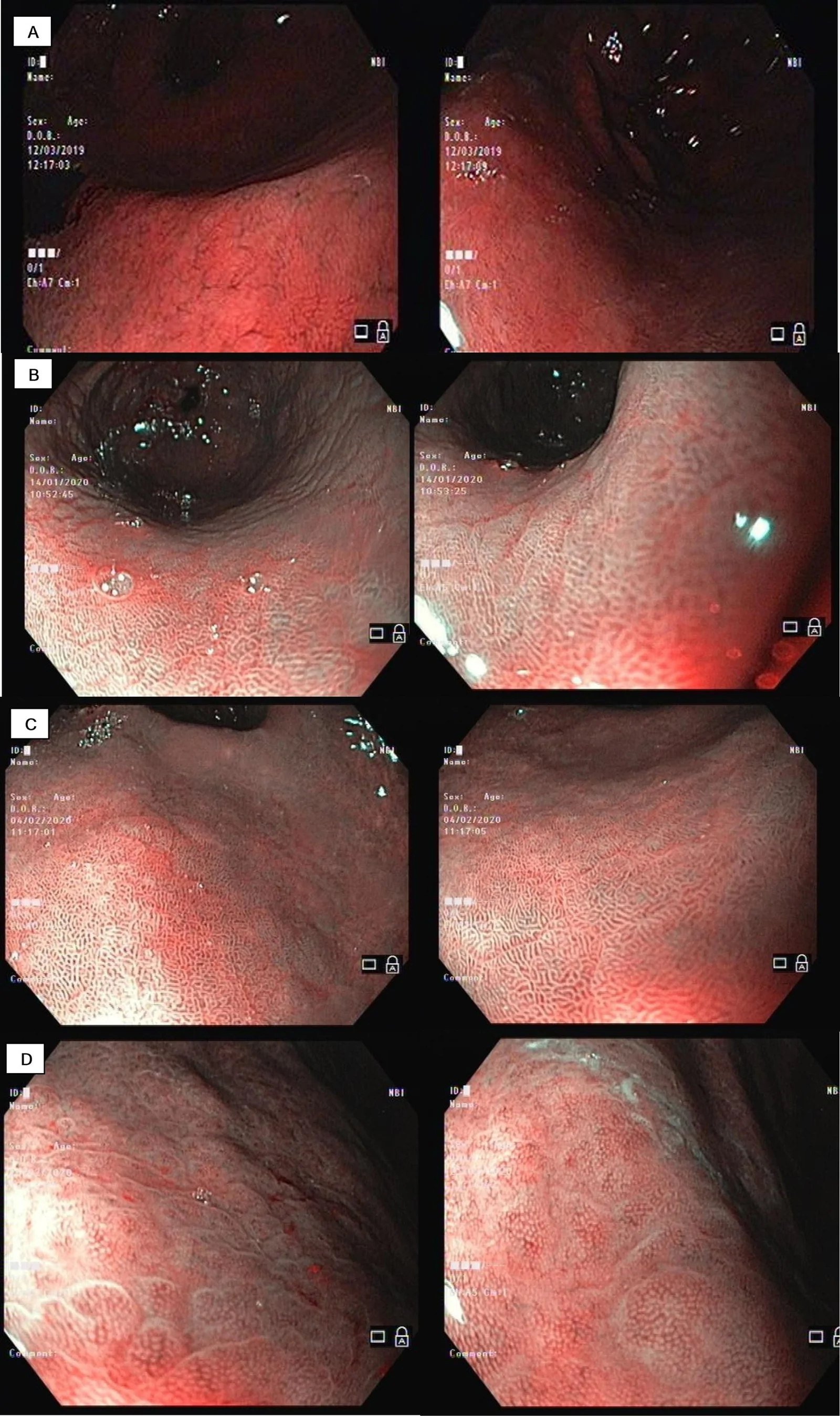
C-NBI mucosal patterns observed: A-Type 1 pattern, B- Type 2 pattern, C- Type 3 pattern, D - Type 4 pattern

###  Biopsy Collection

 Two biopsies were taken from gastric areas demonstrating C-NBI patterns in the following hierarchical order of preference: type 5 > type 4 > type 3 > type 2 > type 1.

 In total, four biopsy samples were taken:

- Two using standard WLE.
- Two using C-NBI endoscopy

###  Rapid Urease Test

 All the obtained biopsies were tested using RUT to detect *H. pylori*. Biopsies obtained using normal WLE and tested with RUT were recorded as conventional RUT. Biopsies taken using C-NBI guided imaging and tested with RUT were recorded as C-NBI RUT. The RUT was done using the Strong Helicotec RUT Plus kit, which is a pH-based colorimetric test that detects the urease activity of *H. pylori* by the color change in the pH indicator in the test.

 Procedure:

1. The adhesive peel was removed from the back of the test slide.
2. The biopsy specimen was transferred onto the well of the test paper with an applicator.
3. The adhesive peel was resealed and was pressed against the test paper to squeeze the tissue fluid out of the biopsy specimen.
4. Patient details and test initiation time were recorded.
5. The outer ring of the test paper was observed for any color change.
6. Color change of the pH indicator from yellow to red indicated the positive presence of *H. pylori,* while the absence of a color change denoted a negative result.

###  Statistical Analysis

 The collected data were coded and entered into the IBM SPSS software (version 20.0). Categorical variables were expressed as numbers and percentages. Associations between variables were analyzed using the Chi-square test and Fisher’s exact test. A P value of less than 0.05 was considered statistically significant.

## Results

 A total of 185 patients with a mean age of 48.97 ± 15.42 years were enrolled in this study. Of these, 123 (66.49%) were men, and 62 (33.51%) were women. Most participants were in the ≥ 60 age group (30.27%).

 At the time of endoscopy, 175 (94.59%) patients were on PPIs, while 10 (5.41%) patients were not taking them. There was no significant association found between the use of PPIs and the results of either Conventional RUT or C-NBI RUT. The small sample size of the non-PPI group (n = 10) limits the statistical power to detect a significant difference. Additionally, 51 (27.57%) patients reported an intake of NSAIDs, while 134 (72.34%) were not on NSAIDs. In the 51 patients taking NSAIDS, 35 patients exhibited erosions and ulcers. This demonstrated a statistically significant association (*P* = 0.01).

###  Conventional RUT Versus C-NBI-RUT

Table 1 summarizes the comparison between conventional RUT and C-NBI RUT. There were three patients who had positive C-NBI RUT but negative conventional RUT, while six patients had negative C-NBI RUT but positive conventional RUT. However, an overall statistically significant association was found between the results of conventional RUT and C-NBI RUT (*P* < 0.001). Furthermore, agreement statistics using Cohen’s kappa yielded k = 0.58, indicating a statistically significant, moderate strength of agreement between the two methods.

**Table 1:** Agreement and positivity yield between conventional RUT and C-NBI-RUT

**C-NBI RUT**	**Positive Conventional - RUT (%)**	**Negative Conventional - RUT (%)**	**Total**	***P*** ** value**
Positive	169 (96.6%)	3 (30.0%)	172 (93.0%)	< 0.001
Negative	6 (3.4%)	7 (70.0%)	13 (7.0%)	
Total	175 (100%)	10 (100%)	185 (100%)	

 Data are presented using frequencies and percentages. The association between methods was analyzed using Fisher’s exact test (*P* < 0.001). Inter-diagnostic agreement was evaluated using Cohen’s kappa statistic, which showed moderate agreement (*k* = 0.58, *P* < 0.001).

###  C-NBI Finding and Results of C-NBI RUT

 Out of the five patterns of C-NBI, Type-2 was the most common type found, followed by Type-4, as seen in Figure 2. Type 5 pattern was not observed. The detection rates of *H. pylori* with RUT among the different types of C-NBI pattern were observed in the following order: Type 3 > Type 2 > Type 4 > Type 1; However, the association between C-NBI patterns and positive C-NBI-RUT was not statistically significant (*P* = 0.378).

**Figure 2:**
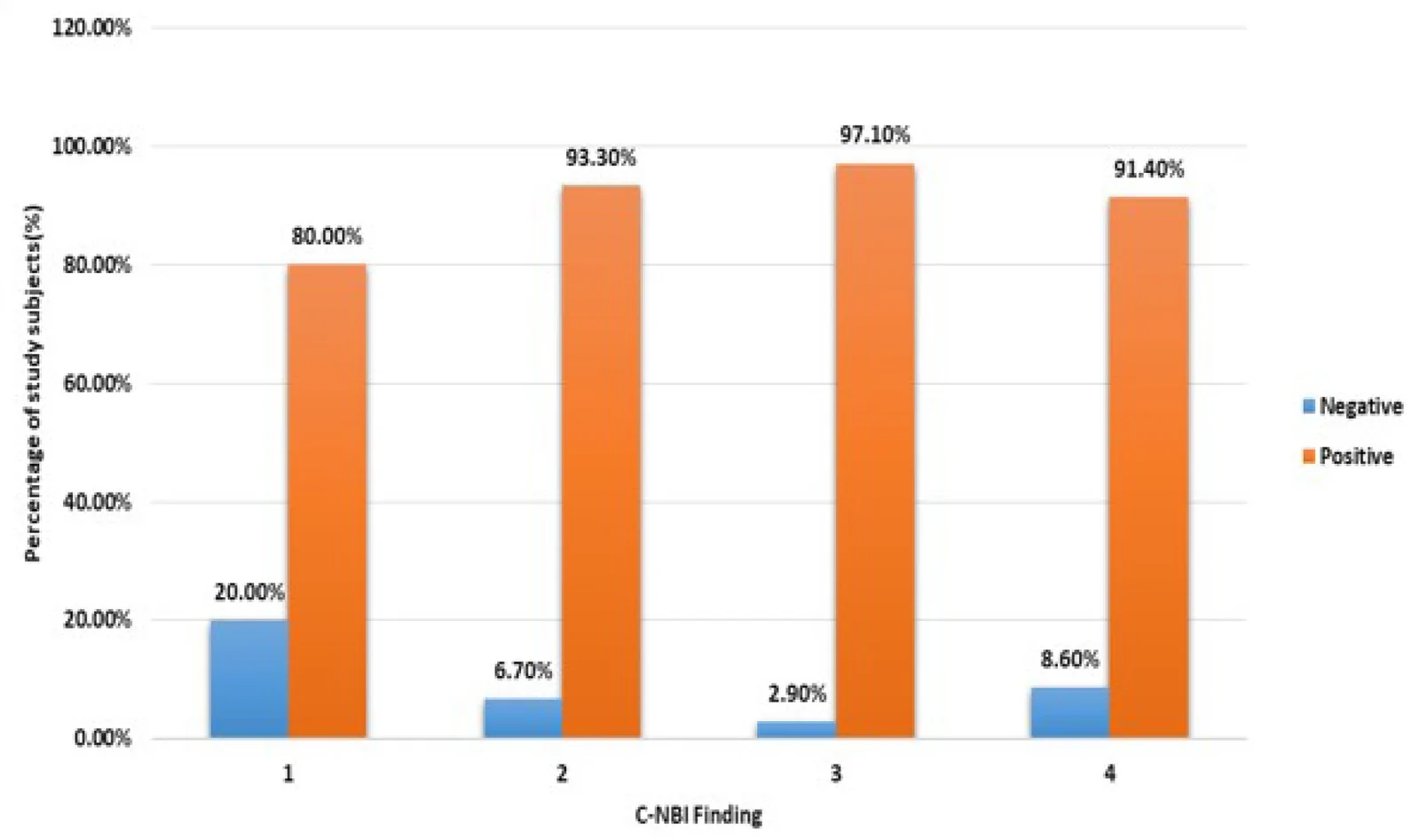
Graph depicting C-NBI findings and results of C-NBI-RUT

###  Upper Gastrointestinal Endoscopic Findings

 Upper gastrointestinal endoscopic findings are summarized in Table 2. Non-erosive pangastritis was the most common finding, followed by non-erosive antral gastritis. In patients with erosive gastritis, lesions were mostly found in the gastric antrum.

**Table 2:** Overall upper gastrointestinal scopy findings

**Overall upper gastrointestinal scopy findings**		**Number (%)**
Non-erosive gastritis	Non-erosive pangastritis	57 (30.81%)
	Non-erosive gastritis of the fundus	1 (0.54%)
	Non-erosive gastritis of the body	21 (11.35%)
	Non-erosive antral gastritis	47 (25.41%)
Erosive gastritis	Erosive pangastritis	3 (1.62%)
	Erosive gastritis of the body	3 (1.62%)
	Erosive antral gastritis	18 (9.73%)
Ulcers	Stricture-D1+D2	2 (1.08%)
	D2-Ulcer	1 (0.54%)
	D1-Ulcer	4 (2.16%)
	Ulcer of the body	3 (1.62%)
	Antral ulcers	6 (3.24%)
Erosions	Erosion of the duodenum	4 (2.16%)
	Erosion of the Fundus	1 (0.54%)
	Erosion of the body	7 (3.78%)
	Antral erosions	7 (3.78%)

 Among peptic ulcer cases, Duodenal ulcer (3.78%) was more common than gastric antral (3.24%) and gastric body (1.62%) ulcers. Distribution of erosions was similar in the gastric antrum (3.78%) and gastric body (3.78%), followed by the duodenum (2.16%).

Table 2 shows the overall gastrointestinal endoscopic findings. Data are presented with descriptive statistics, including frequencies and percentages.

## Discussion

 Dyspepsia is an umbrella term that includes various symptoms originating from the upper gastrointestinal tract and represents a significant healthcare burden worldwide in terms of costs and quality of life.

###  Gender Prevalence of Dyspepsia

 Although dyspepsia is reported to show a higher prevalence in women and smokers,^10^ our study revealed a male predominance, with 123 men reporting dyspepsia compared with 62 affected women. Multiple studies have reported similar findings ^11-14^ and the reason may be attributed to the higher prevalence of upper gastrointestinal tract diseases in men.

###  Prevalence of H. Pylori

 In the present study, a high *H. pylori* prevalence of 94.5% was observed in the study population. A cross-sectional survey done by Shah and colleagues ^15^ showed that 30.4% had dyspepsia, with only 12% of the patients having significant symptoms. The high prevalence in our study may be due to differences in the study population, referral bias commonly seen in tertiary settings, selection bias, and the endemic nature of *H. pylori* infection. Other studies have reported that about 20% of the world’s population suffers from dyspepsia; however, only 10-12% of those patients seek medical care.^10^ A systematic review conducted in 2015 observed that 4.4 billion individuals around the world were projected to be *H. pylori* positive.^16^

 Our study did not show a statistically significant difference in the prevalence of *H. pylori* between the below 50 years and above 50 years age groups. There was no variation in the level of infection based on age, similar to previous studies^14,17,18^. However, among the 20-40-year age group, there was an increase in *H. pylori* infection, which has also been reported by Shokrzadeh and others.^19^ This may be due to lifestyle exposures and higher health-seeking attitudes in this age group.

###  Endoscopic H Pylori Detection

 Technological advancements in endoscopic methods have prompted numerous studies to assess different endoscopic techniques in the clinical diagnosis of *H. pylori*.^20^ In our study, C-NBI was studied using site-specific biopsy. In the present study, conventional RUT(94.6%) showed a marginally higher positivity rate than C-NBI–guided RUT (92.9%) with substantial concordance between the two methods. This was in contrast to previous studies that observed the superiority of NBI in diagnosing* H. pylori-*induced gastritis.^21-23^ Cho et al.^24^ observed that WLE showed a sensitivity of 96.6%, specificity of 59.8%, and accuracy of 72% in diagnosing atrophy, while it was 100%, 59%, and 72%, respectively, for NBI.

###  Correlation of C-NBI Mucosal Patterns with H. Pylori Infection

 In the present study, the Type-2 pattern was commonly seen (75 patients). This pattern was predominantly associated with non-erosive gastritis and showed high *H. pylori* positivity (93.30%). This was markedly higher than that reported by Tongtawee and colleagues,^25^ possibly reflecting differences in the study design and PPI exposure.

 Type-3 (97.1%) and Type-4 (91.40%) patterns demonstrated consistently high *H. pylori* positivity. This is in alignment with existing literature,^25,26^ reinforcing their strong association with active infection. Alaboudy and co-workers,^26^ classified NBI mucosal patterns without magnification and found that Type 3, Type 4, and Type 5 patterns were predominantly *H pylori-*positive.

 Type-1 C-NBI pattern demonstrated variable *H. pylori* association in our cohort, contrasting with prior studies that reported Type-1 mucosa to be predominantly *H. pylori-* negative.^26^ A study by Glover and others ^27^ concluded that the presence of a Type 1 pattern, or regularly arranged collecting venules, helps rule out *H. pylori* infection. This discrepancy may be explained by the high prevalence of PPI use and the inclusion of patients with erosive disease in our study. The absence of the Type-5 pattern in our study suggests a lower prevalence of advanced atrophic changes and *H. pylori*–associated gastric cancer risk in our study population.

###  Distribution of Gastric Lesions

 Gastric antrum was the most commonly involved location (41.62%) compared with the gastric body and fundus, which is similar to existing studies.^21,28^ Nobakht and others ^29^ reported that *H. pylori* is primarily colonized in the antrum, resulting in an antral-predominant gastritis. Among patients with peptic ulcer diseases, duodenal ulcer (3.78%) was more common. Taha et al.^28^ and Paulose et al.^22^ also reported duodenitis as the commonly spotted lesion. Uppin and colleagues ^30^ found a strong association (82.35%) between duodenal ulcers and *H. pylori* in their study.

###  Proton Pump Inhibitors

 Most patients (94.59%) in the present study were on PPIs at the time of endoscopy. This was mainly because they had ongoing symptoms and did not prefer to defer the endoscopy by two weeks. There was no statistically significant difference in the *H. pylori* detection rates between conventional RUT and C-NBI RUT with PPI use. This could be due to the lack of a PPI washout period before the endoscopies. PPI decreases bacterial density and urease activity, leading to false-negative RUT results in both comparison groups. This could have suppressed the potential diagnostic advantages of C-NBI sampling, as noted in other studies, such as one conducted in Thailand, where site-specific biopsy using C-NBI improves identification of *H. pylori* infection in patients after two weeks of PPI cessation.^25^

###  Treatment for Patients with H. Pylori

 Patients identified with *H. pylori* infection were prescribed pantoprazole 40 mg, amoxicillin 1000 mg, and clarithromycin 500 mgtwice daily for 14 days. This was in accordance with the 2017 clinical guidelines by the American College of Gastroenterology (ACG) of PPI-clarithromycin triple therapy.^31^ Patients were further followed up after the 2-week course, and a urea breath test (UBT) was done to confirm eradication of *H. pylori*. Notably, the ACG guidelines in 2024 advise against the use of clarithromycin, considering its low treatment success rates and increased antimicrobial resistance against *H. pylori*. It advises the use of bismuth quadruple therapy (PPI + bismuth + tetracycline + nitroimidazole) as the first line of treatment.^32^ A study in Iran found that a 2-week bismuth quadruple therapy was highly effective, with significant eradication rates across all age groups.^33^

## Strengths and Limitations

 Strengths: The major strength of our study is the use of combined tests for accurate detection of *H. pylori*, which adds to the limited number of studies conducted in the Indian population.

 Limitations: A primary limitation of the study is the lack of an independent reference standard, such as histopathological analysis or qPCR, for detecting *H. pylori*. Consequently, the findings demonstrate only the concordance and sampling yield between the two guided biopsy methods, rather than absolute diagnostic sensitivity and specificity. Another limitation of the study is the lack of an appropriate PPI washout protocol before endoscopy. Almost all participants were on PPIs at the time of the study, a major confounding factor because PPIs reduce bacterial density and RUT sensitivity, leading to false-negative RUT results in both comparison groups. Ambiguous results were found in nine patients, for whom no definitive conclusions could be drawn from the tests. Lastly, the small cohort size and single-center study design limited external validity and generalizability of the results.

## Future Directions

 Multicenter longitudinal studies with large cohorts and standardized PPI washout protocols should be conducted. Results can be compared with gold-standard histopathological references for accurate evaluation. Assessment of inter-observer agreement and cost-effectiveness will clarify the clinical role of C-NBI-guided biopsy in routine practice.

## Conclusion

 C-NBI–guided biopsy showed concordant performance with conventional WLE–guided biopsy in detecting *H. pylori* using the RUT. However, no significant difference in RUT positivity was observed between the two methods in this study population, likely due to the aforementioned limitations
